# A robust NIfTI image authentication framework to ensure reliable and safe diagnosis

**DOI:** 10.7717/peerj-cs.1323

**Published:** 2023-04-21

**Authors:** Shakila Basheer, Kamred Udham Singh, Vandana Sharma, Surbhi Bhatia, Nilesh Pande, Ankit Kumar

**Affiliations:** 1 Department of Information Systems, College of Computer and Information Science, Princess Nourah bint Abdulrahman University, Riyadh, Saudi Arabia; 2 Department of Computer Science and Information Engineering, National Cheng Kung University, Tai-nan, Taiwan, Taiwan; 3 School of Computing, Graphic Era Hill University, Dehradun, India; 4 Amity University, Noida, India; 5 King Faisal University, Al Hasa, Saudi Arabia; 6 School of Technology Pandit Deendayal Energy University Gandhinagar, Gandhinagar, India; 7 GLA University, Mathura, India; 8 Department of Data Science, School of Science, Engineering and Environment, University of Salford, Manchester, United Kingdom

**Keywords:** Watermarking, NIfTI medical image, Affine transform, LWT, Hessenberg matrix decomposition

## Abstract

Advancements in digital medical imaging technologies have significantly impacted the healthcare system. It enables the diagnosis of various diseases through the interpretation of medical images. In addition, telemedicine, including teleradiology, has been a crucial impact on remote medical consultation, especially during the COVID-19 pandemic. However, with the increasing reliance on digital medical images comes the risk of digital media attacks that can compromise the authenticity and ownership of these images. Therefore, it is crucial to develop reliable and secure methods to authenticate these images that are in NIfTI image format. The proposed method in this research involves meticulously integrating a watermark into the slice of the NIfTI image. The Slantlet transform allows modification during insertion, while the Hessenberg matrix decomposition is applied to the LL subband, which retains the most energy of the image. The Affine transform scrambles the watermark before embedding it in the slice. The hybrid combination of these functions has outperformed previous methods, with good trade-offs between security, imperceptibility, and robustness. The performance measures used, such as NC, PSNR, SNR, and SSIM, indicate good results, with PSNR ranging from 60 to 61 dB, image quality index, and NC all close to one. Furthermore, the simulation results have been tested against image processing threats, demonstrating the effectiveness of this method in ensuring the authenticity and ownership of NIfTI images. Thus, the proposed method in this research provides a reliable and secure solution for the authentication of NIfTI images, which can have significant implications in the healthcare industry.

## Introduction

NIfTI (Neuroimaging Informatics Technology Initiative) is a file format used in medical imaging to store and manage image data such as MRIs and CT scans. NIfTI images typically have the extension “.nii” or “.nii.gz” and store data in a way that allows for efficient processing, analysis, and visualization of medical imaging data. The NIfTI format is widely used in medical imaging and neuroscience research and is supported by many software tools for image analysis and visualization. This has made it possible for patients in remote areas to receive specialist care without traveling long distances, reducing the burden on healthcare systems and improving access to care for patients. Additionally, NIfTI images can be easily and quickly transferred over the internet, allowing for real-time analysis and diagnosis by multiple specialists, improving the accuracy and speed of diagnosis.

However, there are also challenges associated with using NIfTI images in telemedicine, such as data privacy, security, and storage concerns. The transfer of sensitive medical data over the internet requires strong security measures to prevent unauthorized access. Data storage and management systems must be reliable and efficient to ensure that images are stored and retrieved efficiently. Despite these challenges, the trend towards telemedicine and using NIfTI images is expected to continue to grow in the coming years as healthcare systems strive to improve access and efficiency while maintaining the highest standards of patient care (Al-Qershi & Khoo, 2011). It highlights the importance of secure and efficient data transfer and storage of NIfTI images in telemedicine. When multiple specialists are working on a patient’s case, they must have access to the same high-quality medical images on time. The transfer and storage of NIfTI images must be secure to ensure patient confidentiality and prevent unauthorized access to sensitive medical information.

Additionally, the increasing demand for telemedicine services during the COVID-19 pandemic has put a strain on healthcare systems and highlighted the need for efficient and effective data management systems. The ability to quickly and accurately access and analyze NIfTI images is crucial in making an accurate diagnosis and determining the best course of treatment for a patient. While using NIfTI images in telemedicine has many benefits, it also poses data security and management challenges. Ensuring that NIfTI images are handled and transferred securely and efficiently is critical in ensuring the accuracy of diagnoses and the best possible outcomes for patients (Xu et al., 2021).

Digital watermarking is an important tool for ensuring the security and safeguarding of the confidentiality of patients’ personal information and medical records when using electronic health records and telemedicine. Its use in medical image authentication and transmission can help to ensure that images are protected, while allowing them to be accessed by authorized personnel for diagnostic purposes, thereby improving the quality and efficiency of healthcare delivery. Watermark ensures the integrity and confidentiality of medical images during transmission. Digital watermarking involves embedding a unique digital signature or identifier into the image data, which can be used to verify its authenticity and prevent tampering or unauthorized access (Zermia et al., 2021).

Digital watermarking of medical photographs can be used to authenticate and transmit such images over the internet safely and efficiently. This helps ensure that medical images are protected and kept confidential while allowing them to be shared and accessed by authorized personnel for diagnostic purposes.

In addition, digital watermarking technologies are also increasingly being used in medical image storage systems to ensure the integrity of images over time and prevent data loss or corruption. By embedding a unique identifier into each image, it is possible to track and manage the images, ensuring that they are stored securely and can be retrieved for diagnostic purposes when needed.

Indeed, the challenge of balancing security and authenticity with image quality is a critical issue in digital watermarking for medical images. The insertion of watermark bits should be done in a way that does not significantly affect the image’s visual quality and does not introduce noticeable distortions or artifacts. One solution to this problem is to use fragile watermarking techniques, which are designed to detect and indicate any tampering or unauthorized modification of the image. This can provide a secure and effective way to verify the authenticity of medical images without introducing persistent distortions. Another solution is to use reversible watermarking techniques, which allow the original image to be restored after the watermark has been removed. This allows medical images to be authenticated without causing any permanent damage or degradation to the image quality. Developing secure and effective digital watermarking techniques for medical images is an ongoing challenge in e-healthcare and telemedicine. To ensure the accuracy of diagnoses and the best possible outcomes for patients, it is important to balance the need for image authenticity and security with high-quality images that can be accurately analyzed and interpreted by medical specialists.

The two main approaches to digital watermarking for medical images are spatial domain and frequency domain methods. Spatial domain watermarking involves directly altering the image’s pixel values, which makes it easier to implement and requires less computing power (Al-Haj & Abdel-Nabi, 2021). However, the downside of spatial domain methods is that they can be vulnerable to image processing attacks and may not provide as robust protection as frequency-domain methods. On the other hand, frequency-domain watermarking involves transforming the image into a different representation, such as a frequency-domain representation using techniques like Fourier Transform (Jhaveri, Patel & Jinwala, 2012), and then incorporating the watermark into the transformed image. This approach is more resilient to image processing attacks and provides more robust protection for medical images. It’s worth noting that each watermarking method has its strengths and weaknesses, and the choice of method will depend on the specific requirements and constraints of the application. In general, frequency-domain (Meena et al., 2022) watermarking is considered more secure and robust and is, therefore, more commonly used in medical image transmission and storage applications.

There have been several studies and research articles that have proposed different digital watermarking methods for medical images. In the article by Meena et al. (2022), a grey watermark is embedded into a grey image using a feature classification forest approach. This method utilizes the characteristics of the image to determine the best location for embedding the watermark and provides a high level of robustness against various types of attacks. Another study suggested a watermarking method based on the integer DCT (discrete cosine transform) domain for embedding various grey watermarks within host grey images. This method utilizes the properties of the DCT transform to provide robust protection for the watermarked images.

The author of the article Li & Zhang (2020) proposed a DWT (discrete wavelet transform) domain-based scheme that combines DCT and SVD (singular value decomposition) the watermark is permanently a part of the medical photos, we need to embed it. Least-squares curve fitting is used to establish the watermark’s insertion strength, which allows for precise control over the watermark strength.

These are just a few examples of the various watermarking methods proposed for medical images (Singh & Singhal, 2018). Each method has its strengths and weaknesses, and the choice of method will depend on the specific requirements and constraints of the application.

A watermarking approach based on SVD matrix decomposition and IWT was proposed by the author Singh & Singhal (2018) (integer wavelet transform). This method involves transforming the image into different sub-bands using IWT and then applying the SVD to create a singular matrix. The watermark bits are embedded into the diagonal values of the singular matrix using a constant embedding strength factor. This method provides robust protection for the watermarked images and allows fine control over the embedding strength. Additionally, IWT and SVD provide high resistance against various image-processing attacks, making this method a promising solution for medical image watermarking.

In the study by Chang & Shen (2017), a hybrid watermarking scheme for grayscale images was presented. The scheme combines the Z transform, DWT, and bidiagonal SVD (BSVD) decomposition. The Arnold Transform was also used to increase the security of the watermark. The procedure begins with a three-level discrete wavelet transform (DWT) that splits the picture into several distinct bands; then, a Z transform is applied to a single band (HL and HH). When the Z-transformed portion has been BSVD’d, the watermark bits are encrypted and embedded in the S matrix’s singular values.

This hybrid approach provides a high level of security for the watermarked images, and the use of the Arnold Transform further increases the security of the watermark. Additionally, combining different transforms provides robustness against various types of image processing attacks, making this method a promising solution for medical image watermarking.

Several approaches for watermarking medical images, including spatial and frequency domain techniques, are used for authentication. Various methods have been proposed, including using features classification forest approach, integer DCT domain (Singh & Bhatnagar, 2018), DWT domain combined with DCT and SVD, IWT with SVD matrix decomposition, a hybrid of Z transform and DWT, and hybrid of DWT and DCT with SVD decomposition. The choice of method will depend on the specific requirements and goals of the medical image authentication protocol, such as resilience, security, and computational effort.

The author’s contribution to the field of medical image watermarking may be seen in the form of a research article given at Kang et al. (2018) that focuses on designing a watermarking strategy tailored to NIfTI pictures. There have not been many studies done on watermarking NIfTI pictures, while most existing systems are built for standard image formats like .jpg, .png, or .bmp, and DICOM images. Modern medical imaging procedures, such as CT scans and MRIs, rely heavily on NIfTI pictures; as such, it is crucial to safeguard their originality and integrity. Developing a watermarking solution for NIfTI pictures necessitates a distinct strategy due to their unique file format when compared to DICOM images. Therefore, your proposed research has the potential to fill the gap in the existing literature on watermarking NIfTI images and provide a reliable and secure solution for authenticating these images. One critical aspect of this research is the need to maintain image quality, as NIfTI images are often used for diagnostic purposes, and any loss of image quality could have severe consequences for patients.

Hsu & Hu (2017) on Protecting copyrighted media in distributed settings is the focus of this research, which introduces a blind watermarking system to do just that. The suggested method strives for stealth, durability, and enough payload capacity.

The method modifies the crisscross discrete cosine transform (DCT) based inter-block to include mean modulation that has been partially sign-altered, as well as mixed modulation. By replacing a single coefficient with a series of them, this method makes the system more resistant to malicious assaults. The use of mixed modulation allows for the regulation of the settings necessary to offer protection against frequently used assaults maintaining a high signal-to-background-noise ratio at the peak.

Singh & Singh (2017) presents a novel method of copyright protection using watermarking based on DWT-SVD and DCT encrypted using the Arnold Cat Map. Typical vulnerabilities in SVD-based watermarking techniques, such as unauthorized reading and false-positive detection, are solved in this method. Moreover, it enhances the system’s reliability and integrity.

The MSB and LSB of each pixel are separated into two sections in the proposed approach, creating a watermark for the grey picture. The DCT coefficients of the middle singular value of blocks 4 by 4 are included in the one-level DWT sub-bands of the host picture. Using Arnold Cat Map as part of the technique, the watermark image may be encoded before being embedded in the host picture for further protection.

Integer wavelet transform (IWT) and singular value decomposition are used in this article to offer a unique method for protecting intellectual property in digital photos, which is reviewed in detail in the next section (SVD). To use this technique, the grey image watermark pixel values are instantly augmented by the singular values of the 1-level IWT decomposition sub-bands. The proposed approach benefits from IWT and SVD’s inherent strengths, making it robust, undetectable, and able to handle a large amount of data.

Area of interest (ROI) and non-region of interest (NII) regions make up half of every image, as stated by Jayashree & Bhuvaneswaran (2019) (NROI). Finally, the watermark is embedded into the NROI by first applying the SLT to 8 × 8 blocks of the NROI and then the SVD to the LH sub-band. SVD’s one-of-a-kind single values are where the watermark is embedded. Use this method to spot tampered photos and prevent others from using yours without permission. Rayachoti, Tirumalasetty & Prathipati (2020) suggest a method in which the NROI is also utilized for watermark insertion. The NROI blocks undergo the Fast Walsh Transform (FWT), and then the SVD is used to embed the first watermark. To embed the second watermark into the red channel of the picture, the SLT transform is utilized. This method allows for the simultaneous insertion of numerous watermarks into a single picture. The SLT and SVD are used in both methods to create watermarks that are secure and resistant to common image processing assaults.

Therefore, ensuring that your watermarking approach does not compromise image quality while providing robust authentication is crucial.

Overall, the proposed research has the potential to significantly impact medical imaging by providing a secure and reliable watermarking solution for NIfTI images.

1. It is designed explicitly for NIfTI images, which are commonly used in medical imaging but have not been extensively studied in watermarking.
2. It uses LWT decomposition to extract precise information from the image, allowing optimal modification during watermark embedding.
3. It applies HD to the LL sub-band, the low-frequency portion of the image, ensuring that the watermark is embedded in an area less likely to be affected by noise or other distortions.
4. It uses an affine transform to insert the watermark into the upper triangular matrix of the Hessenberg matrix decomposition, which provides a highly secure and robust watermarking technique.
5. The proposed method is evaluated using objective metrics such as PSNR, SSIM, and NC, demonstrating its effectiveness in watermark invisibility and attack robustness.

Overall, this research proposes a highly effective and novel watermarking method for NIfTI images that can help authenticate neuroimaging images of lung CT scans and ensure their integrity in the medical system.

## Background theory

### Slantlet transform

The Slantlet transform (SLT) (Rayachoti, Tirumalasetty & Prathipati, 2020; Bamal & Kasana, 2019) is a type of wavelet transform that Ivan Selesnick first introduced in 1998. It is a related version of the discrete wavelet transform (DWT), but with some important differences in its properties and implementation. One of the main advantages of the SLT over the DWT is its improved localization and smoothness properties. This means that the SLT can capture local features and details in a signal or image more accurately than the DWT, and produce a smoother representation of the data. The SLT also can control two zeros in addition to discrete time, which can be useful for certain applications. To implement the SLT, a parallel filter bank structure is used instead of the iterative filtering process used in the DWT. This allows the SLT to apply various filters on each scale simultaneously, which can lead to faster and more efficient computations.

In addition, SLT filters have a significantly shorter length compared to discrete wavelets, which can be beneficial for applications where computation and memory resources are limited. The SLT can be applied to one-dimensional and two-dimensional signals, such as audio and images. To transform an image using the SLT, the columns, and rows of the image are transformed separately using the SLT filter banks. The resulting coefficients are arranged in a matrix format, which represents the SLT of the image. The Slantlet transform is expressed as a matrix format.

(1) $$S = SL{T_N}\; s\; SLT_N^T$$

The inverse SLT transform can be obtained by:

(2) $$s = SLT_N^T\; S\; SL{T_N}.$$

Low-low (LL), low-high (LH), high-low (HL), and high-high (HH) sub-bands are created using these coefficients to represent distinct frequency ranges in the picture. According to the statement, the SLT coefficients are split into four sub-bands, with the lowest frequency components located in the LL sub-band and the highest frequency components in the HH sub-band.

### Hessenberg matrix decomposition

A matrix with orthogonal columns is called an upper Hessenberg matrix, while a matrix with zeros below the first sub-diagonal is called an orthogonal matrix. A square matrix can be broken down into its upper Hessenberg matrix and an orthogonal matrix using the Hessenberg matrix decomposition (Jhaveri & Patel, 2017). The Hessenberg decomposition takes a square matrix A of dimension nn and returns two matrices, Q and H such that

(3) $$A = Q*H*{Q^T}$$

the higher Hessenberg matrix H is multiplied by the orthogonal matrix Q. More specifically, the H matrix takes the following shape:

H = h[1][1] h[1][2] h[1][3] … h[1][n]

h[2][1] h[2][2] h[2][3] … h[2][n−1]

0 h[3][2] h[3][3] … h[3][n−1]

… … …

0 0 0 … h[n][n−1]

0 0 0 … h[n][n]

where h[i][j] is the element in the i^th^ row and j^th^ column of the H matrix. The first column of H is the first column of A, and each subsequent column is generated by applying a series of Householder transformations to the corresponding column of A to eliminate the elements below the sub-diagonal. The Q matrix is the product of these Householder transformations, and it has the property that ${Q^T}Q = Q{Q^T} = I$, where I is the identity matrix.

In this Eq. (3), $Q$ is an orthogonal matrix, and $H$ is the upper Hessenberg matrix, which is constructed using the Householder matrices. For example, consider the following image block $A$ of size $4 \times 4$ and its Hessenberg decomposition.

$A = \left[ {\matrix{ {130} & {245} & {128} & {134} \cr {113} & {226} & {194} & {231} \cr {124} & {212} & {136} & {245} \cr {160} & {189} & {227} & {145} \cr } } \right].$

Utilizing the equation mentioned above, carry out the process of Hessenberg decomposition on the matrix.

$Q = \left[ {\matrix{ {1.000} & 0 & 0 & 0 \cr 0 & { - 0.4874{\rm \; \; \; }} & { - 0.6850} & { - 0.5415} \cr 0 & { - 0.5349} & { - 0.2559} & {0.8052} \cr 0 & { - 0.6902} & {{\rm \; }0.6821} & { - 0.2417} \cr } } \right]$

$H = \left[ {\matrix{ {130.0000} & { - 280.3653{\rm \; }} & { - 109.1788} & { - 61.9701} \cr { - 231.8297} & {583.0474} & {90.9417} & {13.8597} \cr 0 & {130.1586} & { - 25.0484} & {62.5328} \cr 0 & 0 & {36.4188} & { - 50.9990} \cr } } \right]$

$\; {Q^T} = \left[ {\matrix{ {1.000} & 0 & 0 & 0 \cr 0 & { - 0.4874{\rm \; \; \; }} & { - 0.5349} & { - 0.6902} \cr 0 & { - 0.6850} & {{\rm \; } - 0.2559} & {0.6821} \cr 0 & {{\rm \; } - 0.5415} & {0.8052} & { - 0.2417} \cr } } \right].$

### Affine transformation

An affine transformation is a geometric transformation that preserves points, straight lines, and parallel lines, but allows for changes in the size, orientation, and position of objects. Affine transformations can be used to rotate, scale, translate, and skew an object. In image processing, affine transformations are commonly used to modify the position, orientation, and size of an image. This can be useful for tasks such as image registration, where two images are aligned to each other, or for correcting distortions in an image due to the lens or camera imperfections. An affine transformation can be represented by a matrix, which specifies how the transformation is applied to each point in the image. The matrix includes parameters for translation (moving the image), scaling (resizing the image), rotation (rotating the image), and skewing (deforming the image). Affine transformations are a powerful tool in image processing, as they can be used to modify images in a variety of ways while preserving the important features of the image. It is referred to as Eq. (4)

(4) $$\left( {\matrix{ {{H}^{\prime}} \cr {{W}^{\prime}} \cr } } \right) = \left\{ {\matrix{ {S \times \left( {\matrix{ H \cr W \cr } } \right) + \left( {\matrix{ a \cr b \cr } } \right),\; \; if\; H < W - 1} \cr {S \times \left( {\matrix{ H \cr W \cr } } \right) + \left( {\matrix{ 1 \cr b \cr } } \right),\; \; if\; H \ge W - 1} \cr } } \right.$$

The original pixel’s position is represented by $\left( {\matrix{H \cr W \cr } } \right)$ and the encrypted position is represented by $\left( {\matrix{ {{H}^{\prime}} \cr {{W}^{\prime}} \cr } } \right)$. Translation transformations are represented by $\left( {\matrix{ a \cr b \cr } } \right)$ and $\left( {\matrix{ 1 \cr b \cr } } \right)$. Where matrix $S$ is the affine matrix. The affine transform matrix, $S$ is a nonsingular matrix, and its inverse matrix is assumed to be ${S^{ - 1}}$, where $T$ represents the length of the image. The inverse affine transform equation is written as follows: (5)

(5) $$\left( {\matrix{ H \cr W \cr } } \right) = \left\{ {\matrix{ {{S^{ - 1}} \times \left( {\matrix{ {{H}^{\prime}} \cr {{W}^{\prime}} \cr } } \right) - {S^{ - 1}} \times \left( {\matrix{ a \cr b \cr } } \right),\; \; if\; {H}^{\prime} + {W}^{\prime} \le T + 1} \cr {{S^{ - 1}} \times \left( {\matrix{ {{H}^{\prime}} \cr {{W}^{\prime}} \cr } } \right) - {S^{ - 1}} \times \left( {\matrix{ 1 \cr b \cr } } \right),\; \; if\; {H}^{\prime} + {W}^{\prime} > T + 1} \cr } } \right.$$

## Proposed watermarking scheme

In the watermark embedding algorithm will explain how the watermark embedding technique works using the LWT and Hessenberg decomposition methods. In “Watermark extraction algorithm”, we will discuss the watermark extraction procedure, which will be based on the phases of the watermark insertion method that are performed in reverse order.

### Watermark embedding algorithm

First, we safely save the NIfTI picture’s meta-data, and then we pick a section of the image in which to place the watermark. Grayscale pictures are used for both the slice and the watermark. With the use of wavelet transformation, we can break down the NIfTI picture we’ve chosen into four distinct frequency bands. The LL frequency sub-band includes the image’s most important data from being altered by de-noising processes. Perform the Hessenberg decomposition on the LL band. In parallel, the watermark’s main components are subjected to affine transform. Last but not least, the encrypted watermark is added to the Hessenberg decomposition’s H matrix. Reconstructing the image using inverse SLT requires combining the other bands’ components with the adjusted LL frequency sub-bands. In the final step, a watermarked NIfTI image is produced by enhancing the patient’s information (EPR) with the watermarked medical image. The algorithm is described below.

Here is the algorithm for the suggested watermarking technique for NIfTI images:

### Watermark extraction algorithm

Authenticating the NIfTI image watermarking system is used, for this process watermark extraction must be performed. To effectively extract the watermark from a watermarked NIfTI picture, we employ the original NIfTI image in the suggested watermarking method. At first, the NIfTI image and the EPR version of the picture are split apart. The NIfTI image is then watermarked. Next, for each NIfTI picture, select the same slice that was used during watermarking. We next apply the SLT to both the original and watermarked slices, dividing the frequencies into four sub-bands for each. Hence, we can identify which of the two slices contains the watermark. Both LL sub-bands are analyzed using the Hessenberg decomposition now to remove the noise from the H matrix. Affine transformation is applied to each of these noisy parts before assembling the final watermark. These are the measures taken to put a watermark.

**Algorithm 1:** Watermark Embedding Algorithm

**Input:**NIfTI image, watermark image NIfTI image I ( $512 \times 512$), watermark image ₩(64 × 64),
**Output:**Watermarked NIfTI image
**Step-1**Securely save the meta-information of the NIfTI image
**Step-2**Choose a slice X of the NIfTI image for watermark embedding
**Step-3**Convert the selected slice and the watermark image into grayscale
**Step-4**Apply wavelet transformation on the selected slice to separate it into four frequency sub-bands: LL, LH, HL, and HH. (6) $SLT \left( X \right) = \left[ \rm LL,\rm \; LH,\rm \; HL,\rm \; HH \right]$
**Step-5**Apply Hessenberg decomposition on the LL sub-band to obtain the H matrix (7) $WH{W^T} = {\rm Hessenberg\; Decomposition\; }\left( {{\rm LL}} \right)$
**Step-6**Apply affine transform on the watermark image to obtain the encrypted watermark (8) ${ {\mathcal {W}}} = AT\left( \mathcal {W} \right)$
**Step-7**Insert the encrypted watermark bits into the upper triangular matrix of the H (9) $${H}^{\prime} = H + \mu {\rm {\mathbb W}}$$ where H is the Hessenberg matrix, ${{\mathbb W}}$represents the encrypted watermark, and *μ* represents the embedding factor.
**Step-8**Combine the modified LL sub-band and the other frequency sub-bands (10) $L{L^{\prime}} = W{H}^{\prime}{W^T}$
**Step-9**Apply inverse Slantlet transform to obtain the watermarked slice
**Step-10**The watermarked NIfTI picture may be obtained by swapping the selected slice with the watermarked slice
**Step-11**Incorporate the patient’s information (EPR) with the watermarked NIfTI image

**Input:** Read the NIfTI image $X$; watermark image and watermarked NIfTI image ${X^*}$;

**Output:** Extracted the watermark image;

**Step-1:** Watermarked and unwatermarked NIfTI photos’ metadata should be extracted. *i.e*., $X$ and ${X^*}$.

**Step-2:** Select the same slice which is used for the watermarking from both NIfTI images $R$ and $\; {R^*}$

**Step-3:** Decomposed both slices $R$ and $\; {R^*}$ using SLT to obtain the LL band.

**Step 4:** LL sub-band of the watermarked image and the original image are subjected to Hessenberg decomposition separately to obtain the H matrix.

**Step-5:** Extract the watermark from the slice that has been watermarked by reversing the embedding procedure, as described in the Eq. (11).

(11) $${\rm {\mathbb W}} = \left( {{H^*} - H} \right)/\mu$$

where, ${H^*}$ and $H$ represent the Hessenberg matrix of the watermarked slice as well as the host slice respectively. $\mu$ has been used to establish the embedding factor, ${\rm {\mathbb W}}$ is used to represent the extracted component in an encrypted form.

**Step 6:** Lastly, the watermark is obtained by using the inverse Affine transform on the extracted components with the key.

The extracted watermark can then be compared with the original watermark to determine the accuracy of the watermarking process. In order for this approach to be successful, the watermark embedding procedure, the watermarked picture, and the extraction algorithm must all be of high quality.

## Experiment results and analysis

To evaluate the efficacy of the suggested watermarking approach for protecting the privacy of patients’ medical records in accordance with professional standards of care, we used NIfTI’s slice from an open database (Singh et al., 2022). This assessment was carried out only for the purposes of future experimental study. MATLAB 2022a, running Windows 11 and an Intel Core i9-12900H CPU at 5 GHz, was used to test the watermarking technique, 32 gigabytes of random-access memory (RAM) at 4,800 MHz and eight gigabytes NVIDIA GeForce RTX 3070 Ti. The NIfTI images that have been examined have a resolution of 512 × 512 pixels, and a watermark in the form of an a.png file was superimposed on them with a resolution of 64 × 64 pixels with 8-bit depth.

### Performance analysis measures

Six different NIfTI images are used to test how imperceptible the watermarking approach that has been suggested is. The following image quality parameters are utilized to evaluate perceptual imperceptibility (Bhatia et al., 2022). PSNR is used to estimate the perceptibility of watermarks, as seen below.

(12) $$MSE = \displaystyle{1 \over {m*n}}\mathop \sum \limits_{i = 0}^m \mathop \sum \limits_{j = 0}^n {\left( {{A_{ij}} - {B_{ij}}} \right)^2}$$

(13) $$PSNR{\rm \; } = 10*{\log _{10}}\displaystyle{{{{\left( {Max} \right)}^2}} \over {\displaystyle{1 \over {m*n}}\mathop \sum \nolimits_{i = 0}^m \mathop \sum \nolimits_{j = 0}^n {{\left( {{A_{ij}} - {B_{ij}}} \right)}^2}}}$$

(14) $$SNR = 10*lo{g_{10}}\displaystyle{{\mathop \sum \nolimits_{i = 1}^n \mathop \sum \nolimits_{j = 1}^m {{\left( {{A_{ij}}} \right)}^2}} \over {\mathop \sum \nolimits_{i = 1}^n \mathop \sum \nolimits_{j = 1}^m {{\left( {{A_{ij}} - {B_{ij}}} \right)}^2}}}$$

The robustness of the proposed system is evaluated by utilizing the normalized correlation. The value of NC is the difference between the unaltered slice, and the computational equation as (15) (Singh & Singh, 2017).

(15) $$NC = \displaystyle{{\mathop \sum \nolimits_{x = 1}^M \mathop \sum \nolimits_{y = 1}^N w\left( {x,y} \right) \times w*\left( {x,y} \right)} \over {\mathop \sum \nolimits_{x = 1}^M \mathop \sum \nolimits_{y = 1}^N {w^2}\left( {x,y} \right)}}$$

The following Eq. (14) defines the universal image quality index Q (Golub & Loan, 1996).

(16) $$Q = \displaystyle{{4{\sigma _{xy}}\bar{x} \bar {y} } \over {\left( {\sigma _x^2 + \sigma _y^2} \right)\left[ {{{\left( {\bar x} \right)}^2} + {{\left( {\bar y} \right)}^2}} \right]}}$$

It is the result of three factors, which are as follows:

(17) $$Q = \displaystyle{{{\sigma _{xy}}} \over {{\sigma _x}{\sigma _y}}} \times \displaystyle{{2\bar x\bar y} \over {{{\left( {\bar x} \right)}^2} + {{\left( {\bar y} \right)}^2}}} \times \displaystyle{{2{\sigma _x}{\sigma _y}} \over {\sigma _x^2 + \sigma _y^2}}$$

The SSIM (structural similarity index measure) is used to compare the watermarked picture to the original one to ascertain how similar they are. If the SSIM value is 1, then the watermarked and original versions of the picture are identical. The following equation may be used to determine this value (18)

(18) $${\rm SSIM}\,(j,k) \displaystyle{{\left( { 2{\varphi _j}{\varphi _k} + {p_1}} \right) \left( {2{\xi _{jk}} + {p_2}} \right)} \over { \left( {\varphi _{j }^2 + \varphi _j^{2 } + {p_1}} \right) \left( {\xi _j^2 + \xi _k^2 + {p_2}} \right)}}$$

### Results and discussion

**Figure 1:**
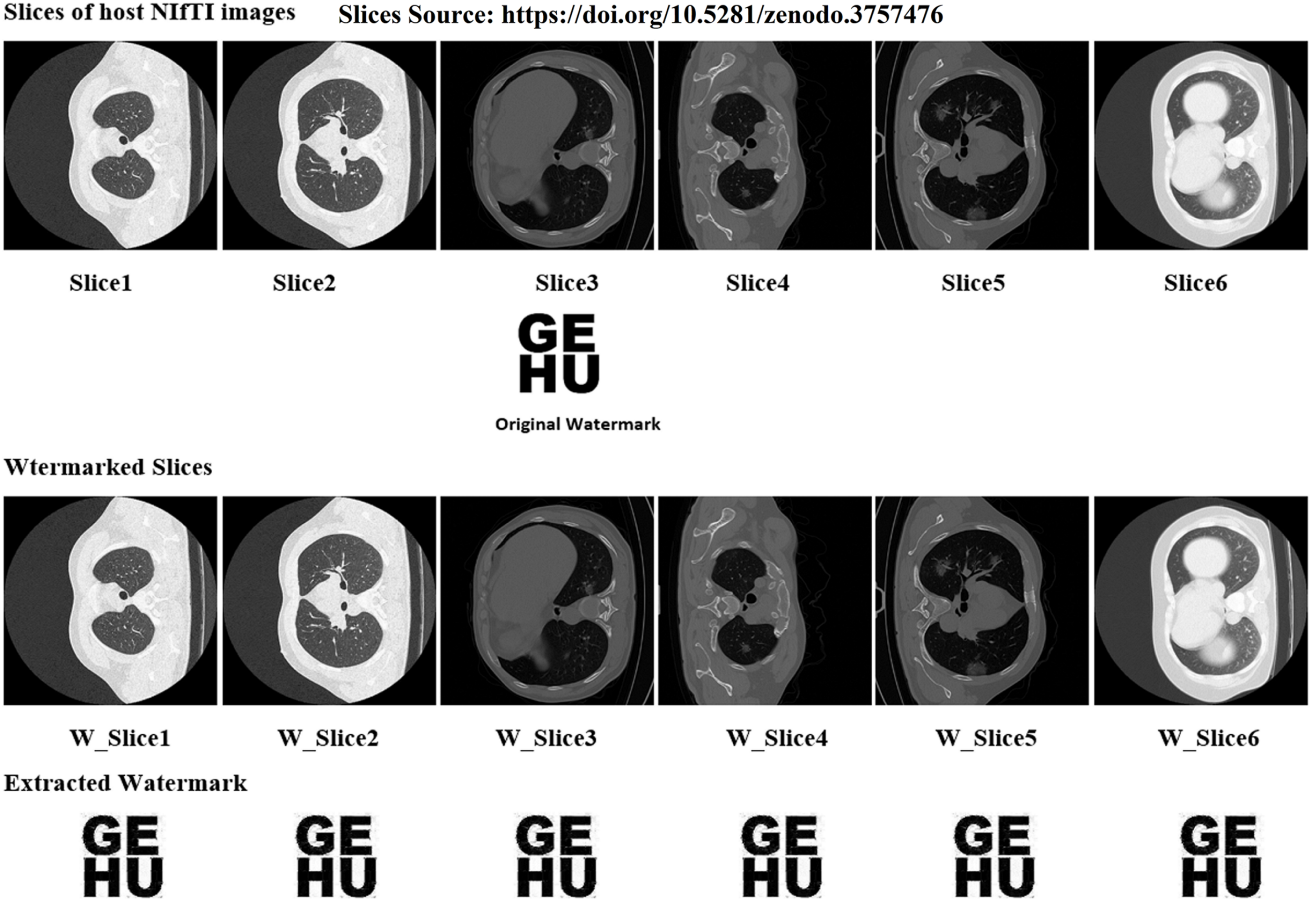
Slices used for the experiment, original watermark image, and extracted watermark. Image source credit: Jun et al. (2020).

**Figure 2:**
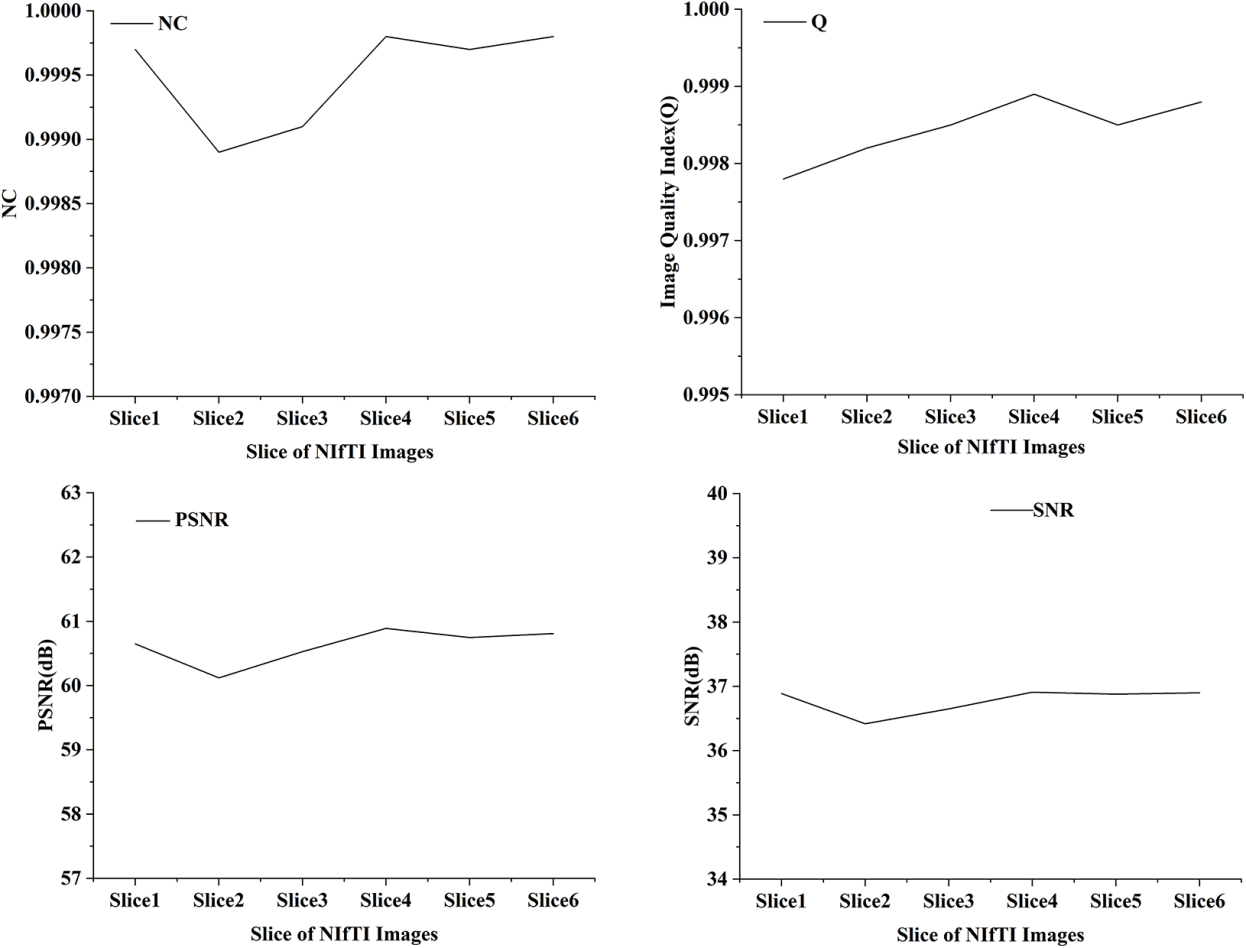
NC, Q, PSNR and SNR results of watermarked slices.

**Figure 3:**
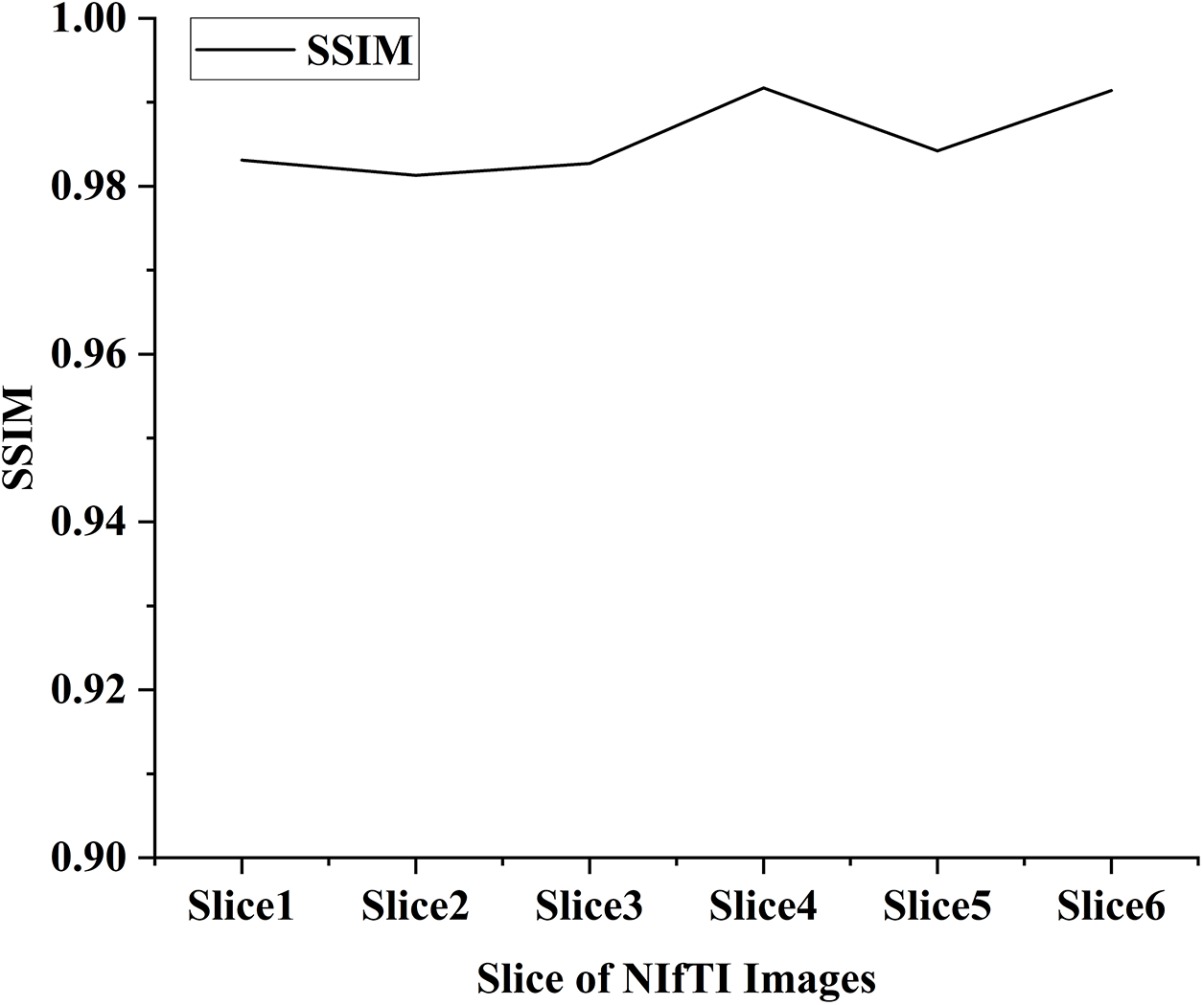
SSIM value of the watermarked slices.

In the proposed work six distinct NIfTI images were used to develop and test the suggested watermarking approach. A 64 × 64 watermark may be created by slicing a 512 × 512 image. Figure 1 and Table 1 display the test results, which demonstrate the watermarking system’s successful imperceptibility and durability. As can be seen in Figs. 2 and 3, the NC, SSIM, and Q values for the images are all very near to 1, showing a high degree of consistency between the unaltered and watermarked versions of the picture. In addition, the PSNR of the watermarked slice is close to 60 dB, suggesting excellent quality. The results of the suggested approach are compared to those of other current methods in Tables 2 and 3, and it shows that the recommended strategy is considerably superior. Taken together, the findings indicate that the custom watermarking technique is a viable method for safeguarding NIfTI photos against theft and redistribution.

**Table 1:** Qualitative analysis of the proposed scheme.

Medical images	PSNR	SNR	SSIM	NC	Q
Slice1	60.65	36.89	0.9831	0.9997	0.9978
Slice2	60.12	36.42	0.9813	0.9989	0.9982
Slice3	60.53	36.65	0.9827	0.9991	0.9985
Slice4	60.89	36.91	0.9917	0.9998	0.9989
Slice5	60.75	36.88	0.9842	0.9997	0.9985
Slice6	60.81	36.90	0.9914	0.9998	0.9988

**Table 2:** Comparison of PSNR with the existing works.

Proposed method	Zermia et al. (2021)	Makbol & Khoo (2014)	Bamal & Kasana (2019)	Kunhu, Al-Ahmad & Taher (2017)	Singh, Singh & Singhal (2018)	Aboden & Agoyi (2018)	Abduldaim, Waleed & Mazher (2020)
60.65	55.850	43.670	55.060	45.830	57.410	40.159	49.900
60.12	57.040		50.270	41.180		40.130	42.740
60.53	44.910		48.990	46.930		40.156	47.100
60.89			47.180	64.930		40.153	46.800
60.75							
60.81							
**Average PSNR**							
60.63	52.600	43.670	50.380	49.780	57.410	40.150	46.640

**Table 3:** Comparison of SSIM with the existing works.

Proposed method	Zermia et al. (2021)	Kunhu, Al-Ahmad & Taher (2017)	Singh, Singh & Singhal (2018)	Aboden & Agoyi (2018)
0.9831	0.998	0.994	0.999	1.000
0.9813	0.998	0.989		1.000
0.9827	0.997	0.999		1.000
0.9917		0.999		1.000
0.9842		0.994		
0.9914		1.000		
**Average SSIM**				
0.986	0.999	0.996	0.999	1

Indeed, watermarking NIfTI images can have significant benefits in the medical field. By adding a watermark to the image, medical professionals and radiologists can ensure that the image they are working with is the ideal medical image and has not been tampered with or altered in any way. This is crucial for accurate diagnoses and can ultimately impact a patient’s life or death. In addition to this, watermarking can also be used to add patient information, such as identification number and name, directly onto the image. This can help in accurate record-keeping and can ensure that the correct patient information is associated with the right image. With the increase in the number of NIfTI images being generated due to COVID-19, the need for accurate and reliable diagnosis has become more important than ever. By implementing watermarking techniques, medical professionals and radiologists can have more confidence in the images they are working with, ultimately leading to more accurate diagnoses and better patient outcomes.

**Figure 4:**
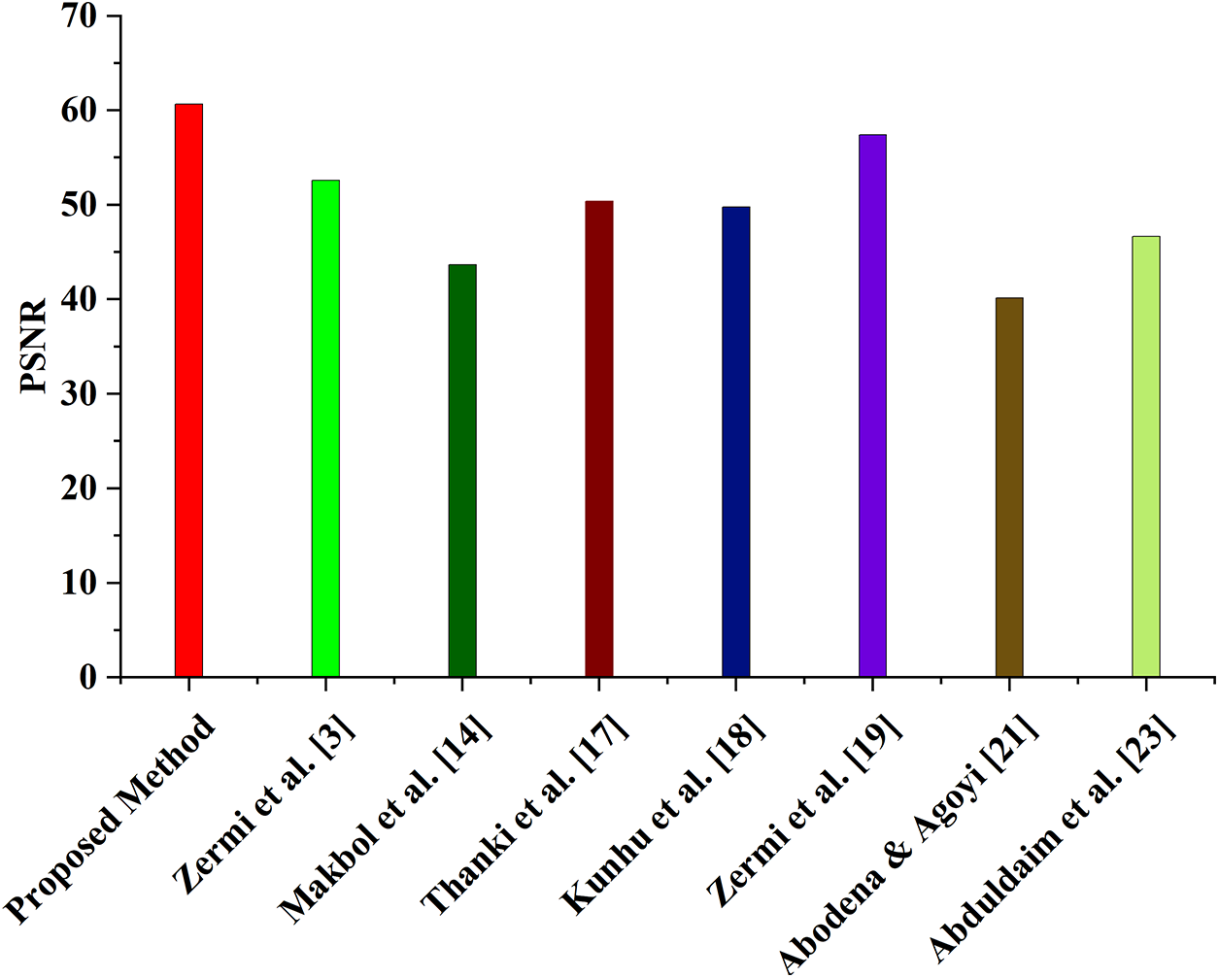
Comparison of average PSNR with the proposed and existing method.

The PSNR values of the proposed watermarking method are compared to those of other current techniques in Table 2 (Zermia et al., 2021; Makbol & Khoo, 2014; Bamal & Kasana, 2019; Kunhu, Al-Ahmad & Taher, 2017; Singh, Singh & Singhal, 2018; Aboden & Agoyi, 2018; Abduldaim, Waleed & Mazher, 2020). Six different NIfTI pictures with PSNR values between 60 and 61 were used to evaluate the suggested approach. The PSNR values of other existing schemes range from 40.13 to 64.93 dB, with an average PSNR of 52.60 dB for scheme (Zermia et al., 2021), 43.67 dB for scheme (Makbol & Khoo, 2014), 50.38 dB for scheme (Bamal & Kasana, 2019), 49.78 dB for scheme (Kunhu, Al-Ahmad & Taher, 2017), 57.41 dB for scheme (Singh, Singh & Singhal, 2018), 40.15 dB for scheme (Aboden & Agoyi, 2018), and 46.64 dB for scheme (Abduldaim, Waleed & Mazher, 2020), as shown in Fig. 4. While some of the existing schemes, such as schemes Zermia et al. (2021), Bamal & Kasana (2019), Kunhu, Al-Ahmad & Taher (2017), Singh, Singh & Singhal (2018), also have a high PSNR, As the suggested approach maintains a high PSNR across all six NIfTI pictures, it is more practical than the other schemes. These results show that the suggested strategy is superior to the alternatives in terms of picture quality and assault resistance.

The proposed scheme has an SSIM value of 0.9903, which is slightly lower than the average SSIM of the existing schemes (Zermia et al., 2021; Kunhu, Al-Ahmad & Taher, 2017; Singh, Singh & Singhal, 2018; Aboden & Agoyi, 2018). Yet, it is still deemed high and demonstrates that the suggested technique can keep picture quality while offering resistance to various image-processing assaults. Using a range from 0 to 1, where 1 denotes complete similarity, the SSIM metric quantifies the degree of structural similarity between two pictures. The greater the structural, textural, and contrast similarity between the photographs, the higher the SSIM score. Results from this study’s SSIM analysis show that the suggested approach can successfully embed and remove watermarks with little quality loss.

### Robustness analysis

The proposed method has been tested by analyzing various slices of the different NIfTI images that were made accessible, and the results produced substantial conclusions. In addition, the method’s durability is assessed by employing image-processing assaults as a part of the evaluation procedure. During this step, the watermarked images are subjected to several different kinds of attacks, including compression, Salt & Pepper, Poisson, compression, speckle noise, and Gaussian noise (Bhatia, 2021; Bharany et al., 2022). When these assaults have been carried out, the watermark will be retrieved from the tempered image and presented in Table 4. The results of the robustness testing that was performed against the various attacks are also included in this table. It was discovered that the results of the robustness testing performed against the various assaults are prominent.

**Table 4:** Watermark extraction after the various attacks.

Name of attack	PSNR of the extracted watermark	SSIM of the extracted watermark	NC of extracted watermark	Extracted watermark
Speckle noise (0.004)	38.33	0.9637	0.9745	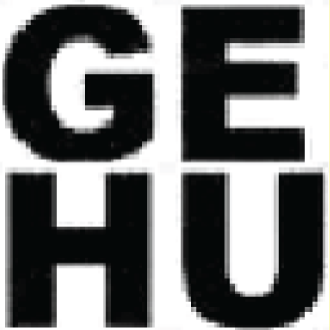
JPEG 80 compression	34.49	0.8533	0.9629	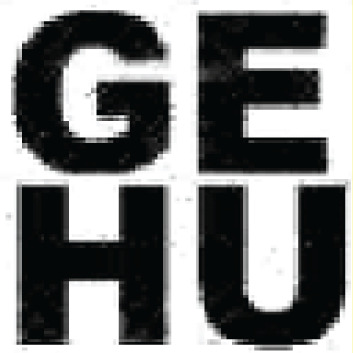
Sharpening (0.2)	32.74	0.9458	0.9531	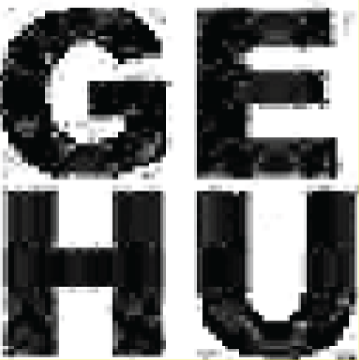
Histogram equalization	28.17	0.8653	0.9289	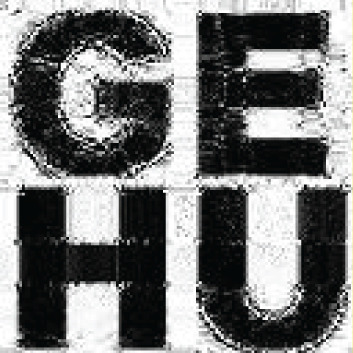
Average filter (3 × 3)	30.74	0.8576	0.9476	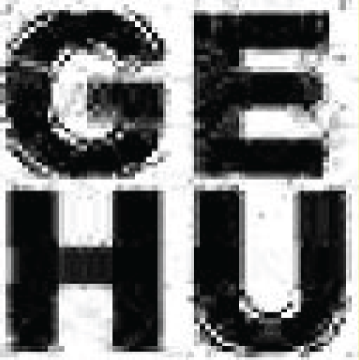
Motion blur (0.2)	27.94	0.7763	0.8859	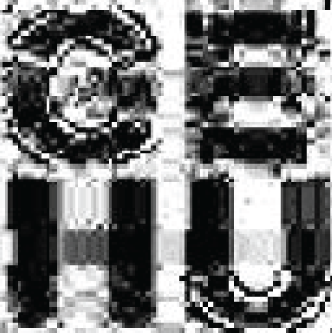
Gaussian low-pass filter (3 × 3)	33.19	0.8631	0.9601	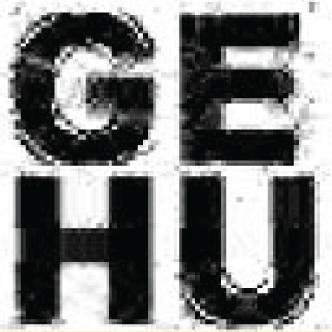
Median (3 × 3)	34.52	0.8587	0.9648	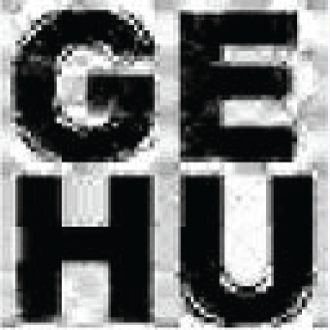
Gaussian noise (0.005)	32.07	0.7167	0.9532	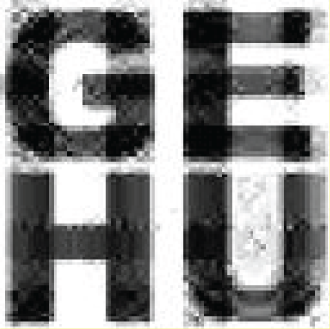
Salt and pepper noise (0.003)	31.87	0.9524	0.9735	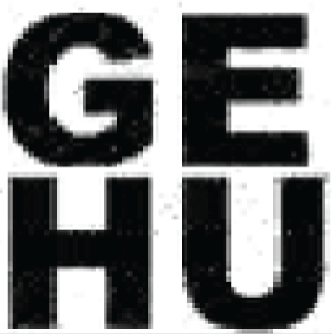

The article explains how the watermarked image can be damaged in numerous ways and the recovered watermark will still be legible. Yet, there is a trade-off to consider, since the approach’s durability and the quality of the watermarked image are inversely related (Singh et al., 2022). For NIfTI pictures generated by CT scan, the suggested watermarking procedure results in a watermarked NIfTI image for safe diagnosis. Because a considerable drop in watermarked picture quality is dangerous and might result in the patient’s death, the suggested approach is evaluated against several noise attacks. The efficiency and dependability of the suggested approach for diagnostic purposes are demonstrated by the quality metrics and parameters indicating that the watermarked picture keeps excellent quality even after being attacked.

## Conclusion

LWT, Hessenberg matrix decomposition, and affine transform are the three approaches proposed in this article as watermarking strategies for NIfTI pictures. The test used a CT scan (512 on 512) to create NIfTI slices, and a grayscale watermark was also used (64 × 64). After incorporating the watermark data into the Hessenberg matrix, an affine transformation was applied to the data. Based on the simulation findings, the suggested technique is superior to alternatives in terms of stealth, robustness, and throughput. A random section of a NIfTI picture was watermarked with a signature to verify its authenticity. Values for NC and SSIM are much closer to 1, suggesting a considerable improvement in scheme quality. The suggested technique yielded a PSNR of 60–61 dB, which is better than the PSNR achieved by previously established methods (40.13 to 64.93 dB). JPEG compression, additive noise, median filtering, and average filtering did not affect the results achieved using the proposed technique. Yet, the watermark could still be extracted from the image, and doing so improved the image’s aesthetic appeal. Experimental findings show that the suggested technique outperforms state-of-the-art methods in terms of distortion level, making it a trustworthy and secure option for certifying NIfTI photos. The technique may be used to check all of the NIfTI image slices. It is also helpful if someone desires to include a watermark in several slices or all slices of the image. Thus, the suggested watermarking technique may be utilized to verify the legitimacy of a medical image and pin down its correct placement in the NIfTI image format for diagnostic purposes.

Before evaluating the watermarking technique in a fully functional PACS (picture archiving and communication system) where medical images are kept and retrieved, operational trials must occur first. For future research, we propose a more effective method for picking regions, and we note that these trials are necessary. Further segmentation strategies will also be utilized to differentiate between return on investment (ROI) and return on net investment (RONI). We are going to make an effort to conduct experiments on a wide variety of NIfTI images.

## Additional Information and Declarations

### Data Availability

The following information was supplied regarding data availability: The code and data are available at Github and Zenodo: https://github.com/kamredudhamsingh/Hess_watermarking; Ma Jun, Ge Cheng, Wang Yixin, An Xingle, Gao Jiantao, Yu Ziqi, Zhang Minqing, Liu Xin, Deng Xueyuan, Cao Shucheng, Wei Hao, Mei Sen, Yang Xiaoyu, Nie Ziwei, Li Chen, Tian Lu, Zhu Yuntao, Zhu Qiongjie, Dong Guoqiang, & He Jian. (2020). COVID-19 CT Lung and Infection Segmentation Dataset (Verson 1.0) [Data set]. Zenodo. https://doi.org/10.5281/zenodo.3757476
